# Transcranial Static Magnetic Field Stimulation over the Primary Motor Cortex Induces Plastic Changes in Cortical Nociceptive Processing

**DOI:** 10.3389/fnhum.2018.00063

**Published:** 2018-02-15

**Authors:** Hikari Kirimoto, Hiroyuki Tamaki, Naufumi Otsuru, Koya Yamashiro, Hideaki Onishi, Ippei Nojima, Antonio Oliviero

**Affiliations:** ^1^Department of Sensorimotor Neuroscience, Graduate School of Biomedical & Health Sciences, Hiroshima University, Hiroshima, Japan; ^2^Institute for Human Movement and Medical Sciences, Niigata University of Health and Welfare, Niigata, Japan; ^3^Human Cortical Physiology and Neurorehabilitation Section, National Institute of Neurological Disorders and Stroke, National Institutes of Health, Bethesda, MD, United States; ^4^FENNSI Group, Hospital Nacional de Parapléjicos, Servicio de Salud de Castilla La Mancha (SESCAM), Toledo, Spain

**Keywords:** transcranial static magnetic field stimulation, non-invasive brain stimulation, intra-epidermal electrical stimulation, nociceptive processing, pain

## Abstract

Transcranial static magnetic field stimulation (tSMS) is a novel and inexpensive, non-invasive brain stimulation (NIBS) technique. Here, we performed non-invasive modulation of intra-epidermal electrical stimulation-evoked potentials (IES-EPs) by applying tSMS or sham stimulation over the primary motor (M1) and somatosensory (S1) cortices in 18 healthy volunteers for 15 min. We recorded EPs after IES before, right after, and 10 min after tSMS. The IES-EP amplitude was significantly reduced immediately after tSMS over M1, whereas tSMS over S1 and sham stimulation did not affect the IES-EP amplitude. Thus, tSMS may affect cortical nociceptive processing. Although the results of intervention for experimental acute pain in healthy subjects cannot be directly translated into the clinical situation, tSMS may be a potentially useful NIBS method for managing chronic pain, in addition to standard of care treatments.

## Introduction

Epidural electrical stimulation of the primary motor cortex (M1) relieves pain (Tsubokawa et al., 1991a,b). Thus, M1 is an important target region for treatments to alleviate chronic pain. A European team of experts recently established guidelines for application of repetitive transcranial magnetic stimulation (rTMS) and transcranial direct current stimulation (tDCS) and cited sufficient, level A evidence (definite efficacy) for the effect of high-frequency (HF) rTMS over M1 to relieve neuropathic pain (see review, Lefaucheur et al., 2014). They also cited level B evidence (probable efficacy) for anodal tDCS over M1 in fibromyalgia, and level C evidence (possible efficacy) for HF rTMS over M1 in complex regional pain syndrome and for anodal tDCS over M1 in chronic lower limb neuropathic pain secondary to spinal cord lesions (see review, Lefaucheur et al., 2017). Although M1 is also the most widely used target for modulation of experimentally provoked pain by non-invasive brain stimulation (NIBS) in healthy subjects, the results differ widely from those observed in patients with chronic pain, and the optimum stimulation target (M1, primary somatosensory cortex (S1), or other sites) and type of stimulation (facilitatory or inhibitory) for modulation of cortical nociceptive processing have not yet been definitively determined. For example, the amplitude of laser evoked potentials (LEPs) is attenuated by HF rTMS over M1 (facilitatory; de Tommaso et al., 2010), continuous theta-burst stimulation (TBS; inhibitory) over both M1 (Csifcsak et al., 2009b) and S1 (Poreisz et al., 2008), intermittent TBS (facilitatory) over S1 (Poreisz et al., 2008), and cathodal tDCS (inhibitory) over both M1 (Terney et al., 2008; Csifcsak et al., 2009a) and S1 (Antal et al., 2008). These conflicting results can be speculated as resulting from the differences in neural networks involved in the processing of acute provoked nociceptive stimuli in healthy subjects vs. chronic pain in patients. Therefore, experimental acute stimuli in healthy volunteers may not represent chronic pain in patients with neurological lesions. However, examination of acute pain in healthy controls could lead to optimization of new NIBS techniques and increase understanding of cortical regulation of nociceptive processing (reviewed in Mylius et al., 2012).

Transcranial static magnetic field stimulation (tSMS) is a new type of NIBS. tSMS reduces cortical excitability using static magnetic fields (SMFs) applied across the scalp with a cylindrical neodymium, iron and boron (NdFeB) permanent magnet (Oliviero et al., 2011). SMFs have a constant intensity and direction over time, a frequency of 0 Hz, and are different from electromagnetic fields that vary over time. SMFs with moderate intensity (1–1000 mT) magnetically reorient membrane phospholipids and ion channels via diamagnetic anisotropy (Rosen, 2003). SMF stimulation induces changes in voltage-gated calcium channels, intracellular calcium flow, and membrane depolarization (Rosen, 1996, 2003; Pall, 2013; Lu et al., 2015; Prasad et al., 2017). Long-term depression is a result of reduced calcium flow and the subsequent increase in intracellular calcium ion levels caused by blockade of calcium channels (Nakano et al., 2004; Paulus, 2011). In line with previous cellular and animal studies, SMFs applied to the human scalp are believed to decrease cortical excitability. In recent works, tSMS over M1 was shown to not only suppress the corticospinal pathway (Oliviero et al., 2011; Silbert et al., 2013), but also to enhance short-latency intracortical inhibition (SICI; Nojima et al., 2015, 2016). Further, we showed that tSMS over S1 decreases the amplitude of the N20 component of somatosensory evoked potentials (SEPs) following median nerve stimulation (Kirimoto et al., 2014) and alters normal somatosensory processing (Carrasco-López et al., 2017). In addition, similar to rTMS (Enomoto et al., 2001), TBS (Ishikawa et al., 2007) and tDCS (Matsunaga et al., 2004), tSMS over M1 decreases the amplitude of the N33 component of SEPs (Kirimoto et al., 2016). Thus, although the focus of tSMS is small, different components of SEPs are decreased depending on the location of tSMS. In addition, tSMS over M1 or S1 may modulate cortical nociceptive processing, similar to other NIBS techniques. However, to the best of our knowledge, these are still open questions.

Several previous researchers have developed and used the method of intraepidermal electrical stimulation (IES) for selective activation of afferent nociceptive fibers, with minimal effect on Aβ fibers, for pain relief in new pain conditions (Inui et al., 2002; Inui and Kakigi, 2012). This method has several advantages: it can activate Aδ and C fibers preferentially with very low intensity stimuli (0.01–0.03 mV), is easy to control, and avoids skin lesions and prolonged pain. Indeed, in a study using concentric planar electrodes (Kaube et al., 2000), which can stimulate superficial skin layers without the use of a needle, all subjects reportedly declared that they would prefer superficial electrical stimulation rather than CO_2_ laser stimulation if they required pain-related evoked potentials again, although the pain sensation with both techniques was equal, ranging from 60 to 70 on a 0–100 mm visual analog scale (VAS; Lefaucheur et al., 2012a). Although numerous studies have used LEPs to assess the brain response to nociceptor activation and to minimize the discomfort and adverse effects accompanying system-specific stimulation for ethical reasons, we believe that less invasive methods for activation of Aδ and C fibers, such as IES or superficial electrical stimulation using a concentric planar electrode, are more suitable for studies that explore the effect of NIBS intervention on nociceptive processing.

Thus, the aim of this study was to determine whether tSMS over M1 or S1 modifies the evoked potentials (EPs) generated following selective stimulation of afferent nociceptive fibers by IES. New NIBS approaches including tSMS are well tolerated, inexpensive, and useful for self-management of pain by patients. Thus, demonstrating that tSMS over M1 and/or S1 affects the amplitude of IES-EPs could be very important.

## Materials and Methods

### Subjects

We studied 18 healthy volunteers (10 males and 8 females, 21–36 years old) who were not receiving medical treatment for any reason. All participants provided written, informed consent prior to the experiment, which was conducted in accordance with the principles of the Declaration of Helsinki. The protocol was also approved by the Ethics Committee of Niigata University of Health and Welfare. All participants were strongly right-handed as determined by an Oldfield inventory score of 0.9–1.0.

### Sample Size Calculation

The formula below was used to calculate the sample size:

$$n\;=\;\frac{\lambda^{2}C^{2}}{e^{\text{2}}}\;=\;17.23$$

where *λ* is the estimated non-centrality parameter (1.96 for the 95% confidence interval), *C* is the coefficient of variance of the amplitudes of pain-related evoked potentials (~0.106) as previously reported (Otsuru et al., 2010), and* e* is the acceptable error rate of 0.05.

### Experimental Procedure

**Figure 1:**
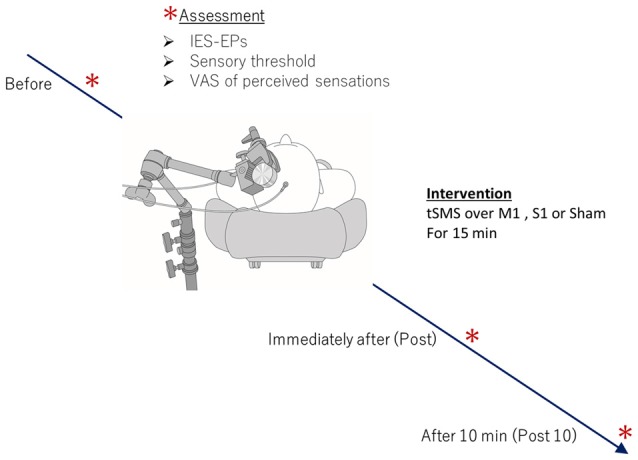
Experimental procedure. During the experiment, subjects were seated in a comfortable reclining armchair with mounted head and arm rests. Eighteen healthy subjects received one transcranial static magnetic field stimulation (tSMS) each over primary motor cortex (M1) and primary somatosensory cortex (S1), and sham stimulation for 15 min in a counter-balanced order. Intra-epidermal electrical stimulation-evoked potential (IES-EP) recordings, sensory threshold measurement, and scoring of visual analog scale (VAS) of perceived sensations were performed before, immediately after, and 10 min after tSMS.

Participants sat in a comfortable recliner with mounted head and arm rests, and all experiments were performed with the forearm in a neutral position. All subjects received one tSMS each over M1 and S1 (real tSMS), as well as sham stimulation, for 15 min in a counter-balanced order. To avoid carryover effects, each volunteer completed three sessions on separate days that were each at least 7 days apart. For recording of nociceptive evoked potentials from the cranial vertex, IES was applied to the dorsum of the right hand using a stainless steel concentric bipolar needle electrode (PNS-7000; Nihon Kohden, Tokyo, Japan) immediately after tSMS/sham stimulation. intra-epidermal electrical stimulation-evoked potential (IES-EP) recordings, sensory threshold measurement and scoring of VAS of perceived sensations were performed before, immediately after, and 10 min after tSMS and sham stimulation (Figure 1).

### tSMS

We used a cylindrical NdFeB neodymium magnet (diameter, 50 mm; height, 30 mm) with a surface magnetic flux density of 534 mT, maximum energy density of 49 MGOe, and a nominal strength of 862 N (NeoMag Co., Ltd., Ichikawa, Japan) for tSMS. We previously showed that this magnet generates a magnetic field that accesses most cortical regions (strength 110–90 mT at 2–3 cm from the surface of the magnet) and elicits biological effects (Kirimoto et al., 2016). Sham stimulation was performed with a non-magnetic stainless steel cylinder that was similar in appearance to the NdFeB magnet (NeoMag Co., Ltd.). The cylindrical magnet or sham device was placed over the participant’s scalp with the aid of an arm-type lightning stand (C-stand, Avenger, Cassola, Italy). To stimulate the left M1, the NdFeB magnet was centered over the region that represents the right first dorsal interosseous (FDI) muscle, which was located with a single TMS pulse. To stimulate the left S1, the magnet was placed 3 cm posterior to the C3 area (C3’) according to the International 10–20 system for electrode placement. tSMS effects are polarity independent (Oliviero et al., 2011), and thus, we used only south polarity for all experiments. Sham stimulation was performed over the left M1 with nine participants and over the left S1 for the other nine. Double blinding was established with two experimenters. The first experimenter chose the intervention (real or sham), placed the device on the participant, and performed the stimulation. The second experimenter, who was blinded to the selection of sham or real stimulation, recorded EPs and analyzed their latencies and amplitudes.

### IES

**Figure 2:**
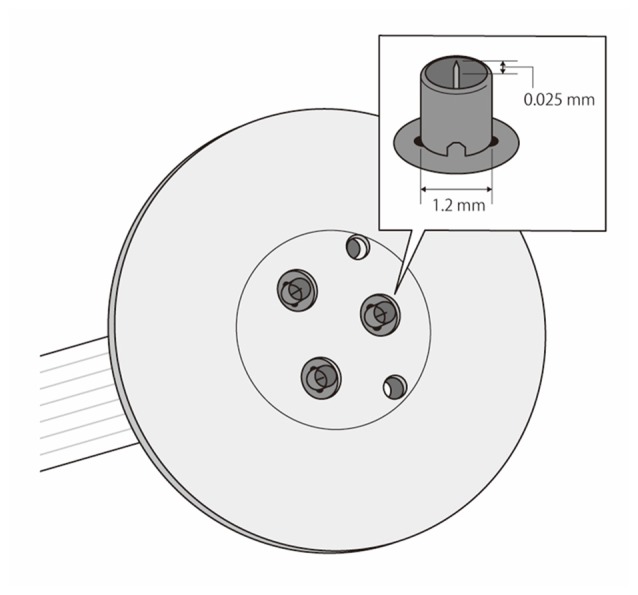
Schema of the stainless steel concentric bipolar needle electrode. We used the method of IES for selective activation of cutaneous Aδ fibers with minimal effects on Aβ fibers with this electrode. The structure was modified to reduce the undesired loop current that reaches deeper skin layers for nociceptive stimulation. The anode was an outer ring 1.2 mm in diameter, and the cathode was an inner needle that protruded 0.025 mm from the outer ring.

For nociceptive stimulation, we performed IES to selectively activate cutaneous Aδ fibers, with little or no activation of Aβ fibers (Inui et al., 2002; Inui and Kakigi, 2012). We used a stimulator specifically designed for IES (PNS-7000; Nihon Kohden) and a stainless steel concentric bipolar needle electrode (NM-980W; Nihon Kohden) that can be changed to decrease the unwanted loop current that reaches deeper skin layers (Mouraux et al., 2010). An outer ring that was 1.2 mm in diameter functioned as the anode, and an inner needle that extended 0.025 mm from the outer ring functioned as the cathode (Figure 2). We gently placed the electrode against the participant’s skin to insert the tip of the needle into the epidermis, which contains the nociceptors. We attached the outer ring to the surface of the skin. The skin above the FDI muscle was washed with alcohol, and we used electrode paste (Gelaid, Nihon Kohden) to decrease electrode impedance. IES was applied to the dorsum of the right hand, approximately over or around the FDI muscle, and the sensory threshold was measured. The parameters of IES to ensure selective stimulation of Aδ fibers included a triangular electric pulse wave with a rise and fall time of 0.5 ms and a train of double pulses with an inter-stimulus interval (ISI) of 10 ms (Kodaira et al., 2014). The intensity of the stimulus for recording EPs was fixed at 1.8–2.0 times the mean value of the sensory threshold described below. Initial stimulation was performed at 0.01 mA and increased in steps of 0.01 mA until the volunteer reported a pricking sensation. Stimulation was then decreased in 0.01-mA steps until the sensation disappeared. The sensation typically disappeared when the stimulus intensity was decreased by 0.01 mA, but a few participants reported a similar albeit weaker sensation at this intensity. We recorded measurements at three locations, because the threshold varied slightly at different depths of penetration, and we used the mean value for analysis (Otsuru et al., 2010). To assess the sensory threshold for IES, volunteers reported the intensity of perceived sensations using the VAS, in which zero meant “no pain” and 10 meant “the most intense pain sensation imaginable”. We asked subjects to indicate the VAS of perceived sensations at a stimulus intensity of 1.8–2.0 times the sensory threshold (the threshold at which no pain, but pricking or tingling sensations occurred in all subjects), which was the stimulus intensity adopted for recording of EPs.

### Measurement of IES-EPs

We performed electroencephalography and recorded IES-EPs as large vertex potentials. Because the maximum response is recorded from the Cz derivation (Kakigi et al., 1989), we recorded evoked responses at Cz. The Cz electrode was considered the active electrode and was referenced to the linked earlobe (A1–A2) as determined by the International 10–20 system of electrode placement using Ag-AgCl electrodes (1.0 cm diameter). We employed a preamplification system (BA1008, Nihon Santeku, Osaka, Japan) to record electroencephalography signals with a bandpass filter of 0.1–100 Hz and a sampling rate of 2000 Hz. The ground electrode was placed on the right forearm using disposable gel electrodes (GE Health Care Japan, Tokyo, Japan). Impedance of the electrodes was kept below 5 kΩ. The IES was presented over the dorsum of the right hand with an ISI of 10–15 s for recording of the IES-EPs, and 12 artifact-free EPs were recorded and averaged. These EPs were recorded prior to tSMS as well as right after, 5 min after, and 10 min after tSMS for 15 min. Analysis was performed with data from 100 ms before beginning IES (considered the DC baseline) to 600 ms after. The skin temperature of the feet was monitored and kept higher than 32°C throughout the examination, by using a non-contact forehead infrared thermometer (DT-8806H, CEM Instruments, West Bengal, India) and regulating the room temperature.

### Data and Statistical Analysis

For Aδ fiber stimulation, the IES-EPs consisted of negative-positive waveforms (N2-P2). The peak latencies of N2 and P2 were approximately 200 and 350 ms, respectively. In addition, we considered the peak latencies of N2 and P2 to be the latency period during 180–250 and 300–400 ms, respectively (Inui et al., 2002; Mouraux et al., 2010; Otsuru et al., 2010; Iwabe et al., 2014; Kodaira et al., 2014; Omori et al., 2017). Amplitudes of EPs were normalized to those recorded before tSMS. Data for the N2 and P2 latencies, normalized amplitudes of IES-EPs (N2-P2), sensory threshold, and VAS for perceived sensations are shown as the mean ± SEM. Two-way repeated-measures analysis of variance (ANOVA) with respect to the tSMS stimulus site (M1 vs. S1 vs. Sham) and time (before vs. right after tSMS vs. 10 min after tSMS) was performed. Bonferroni’s correction for multiple comparisons was used for *post hoc* analysis. *p* < 0.05 was considered statistically significant for all analyses. Statistical analyses were performed using the SPSS Statistical Package, version 21.0 (IBM SPSS).

## Results

**Figure 3:**
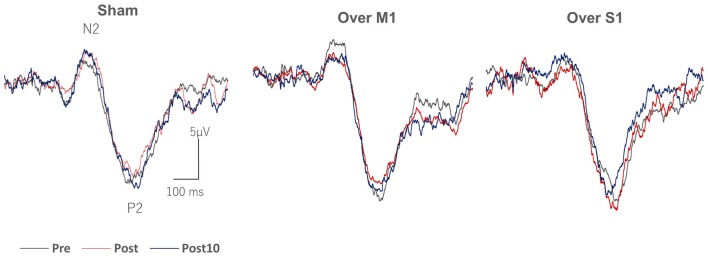
Grand averaged IES-EP waveforms recorded from Cz after tSMS over M1 and somatosensory (S1), and sham stimulation. Note the attenuation of the amplitude of the N2-P2 component immediately and 10 min after tSMS over M1.

Figure 3 shows grand averaged wave forms of IES-EPs (N2-P2) recorded before, immediately after, and 10 min after 15 min of tSMS over M1 and S1, and sham tSMS. The amplitudes of EPs significantly decreased immediately and 10 min after 15 min of tSMS over M1, whereas no overt changes were seen with tSMS over S1 or with sham stimulation. The amplitudes of EPs before each stimulus condition were comparable: sham, 26.2 ± 1.8 μV; tSMS over M1, 26.5 ± 2.0 μV; and tSMS over S1, 25.4 ± 1.7 μV, respectively (*p* > 0.05).

**Figure 4:**
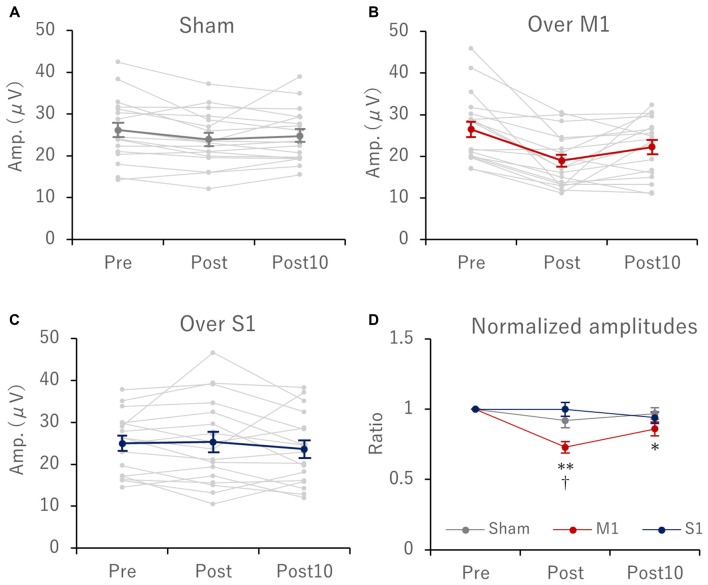
Serial changes in IES-EP amplitudes before (pre), immediately (post), and 10 min (post 10) after tSMS for 15 min. Scatter plots showing the individual (gray lines) and mean (red line) value with each stimulation condition: **(A)** sham, **(B)** M1 and **(C)** S1 stimulation. **(D)** shows serial changes in normalized IES-EP amplitudes with respect to those recorded before tSMS. With M1 stimulation, the amplitudes of EPs were significantly reduced immediately (27 ± 0.04%, *p* < 0.0001) and 10 min (14 ± 0.05%, *p* = 0.045) after tSMS. In addition, immediately after tSMS over M1, the amplitude of EPs was significantly decreased compared with after sham stimulation (*p* < 0.0001) and tSMS over S1 (*p* = 0.002; Panel **(D)**; *p < 0.05, **p < 0.0001 vs. pre, ^†^*p* < 0.01 vs. S1 and sham stimulation).

Serial changes in mean and individual amplitudes of EPs before, immediately after, and 10 min after tSMS at each stimulation condition (sham, and tSMS over M1 and S1) are summarized in Figures 4A–C, respectively. For the normalized amplitudes of EPs, two-way repeated-measures ANOVA revealed a significant main effect of stimulation site (*F*_(2,34)_ = 7.61, *p* = 0.002, $\eta_{\text{p}}^{2}$ = 0.309), time (*F*_(2,34)_ = 11.669, *p* < 0.0001, $\eta_{\text{p}}^{2}$ = 0.407), and interaction between stimulation site and time (*F*_(4,68)_ = 4.514, *p* = 0.003, $\eta_{\text{p}}^{2}$ = 0.21). With M1 stimulation, the amplitudes of EPs were significantly reduced immediately (27 ± 0.04%, *p* < 0.0001) and 10 min (14 ± 0.05%, *p* = 0.045) after tSMS. In addition, immediately after tSMS over M1, the amplitude of EPs was significantly decreased compared with after sham stimulation (*p* < 0.0001) and after tSMS over S1 (*p* = 0.002; Figure 4D). On the other hand, under all tSMS conditions, we observed no remarkable effects on the latency of N2 and P2, sensory threshold, or VAS scores of perceived sensations (Table 1).

**Table 1:** Evoked potential values, sensory threshold and visual analog scale (VAS) scores of perceived sensations with each stimulus condition.

		N2 Latency (ms)	P2 Latency (ms)	N2-P2 Amp. (μV)	Threshold (mA)	VAS (points)
Sham	Pre	226.6 ± 8.2	328.2 ± 10.9	26.2 ± 1.9	0.07 ± 0.01	1.11 ± 0.08
	Post	225.1 ± 7.4	329.2 ± 12.5	23.9 ± 1.6	0.07 ± 0.01	1.06 ± 0.06
	Post10	228.8 ± 8.8	339.8 ± 10.8	24.8 ± 1.6	0.07 ± 0.01	1.11 ± 0.08
M1	Pre	232.1 ± 7.5	349.8 ± 10.5	26.5 ± 2.0	0.08 ± 0.01	1.17 ± 0.09
	Post	236.7 ± 17.1	334.4 ± 12.3	18.9 ± 1.6	0.07 ± 0.01	1.06 ± 0.06
	Post10	239.1 ± 9.8	338.0 ± 11.8	22.2 ± 1.7	0.07 ± 0.01	1.06 ± 0.06
S1	Pre	232.4 ± 10.0	331.7 ± 9.8	25.0 ± 1.9	0.08 ± 0.01	1.06 ± 0.06
	Post	224.2 ± 18.6	333.2 ± 22.7	25.3 ± 2.7	0.08 ± 0.01	1.11 ± 0.08
	Post10	228.1 ± 10.1	306.3 ± 21.3	23.6 ± 2.2	0.07 ± 0.01	1.06 ± 0.06

## Discussion

This study demonstrated that the amplitude of IES-EPs (N2-P2) decreased significantly by up to 15%–25% immediately and 10 min after a 15-min period of tSMS over M1. In contrast, the amplitude of IES-EPs did not show overt changes with tSMS over S1 or sham stimulation. No significant effect on the sensory threshold or VAS of perceived pain sensations was observed under any tSMS conditions.

### IES-EPs

In this study, the parameters for selective stimulation of Aδ fibers for recording EPs were based on the experimental protocol of our previous studies, which involves using trains of double pulses with an ISI of 10 ms at an intensity that was approximately twice the sensory threshold (Otsuru et al., 2010). The values of EP parameters recorded, such as the latency of N2 and P2, amplitudes of EPs (N2-P2), and sensory threshold, were within the range of the results of our and another group’s studies, which employed similar common parameters (Mouraux et al., 2010, 2014; Omori et al., 2013, 2017; Iwabe et al., 2014; Kodaira et al., 2014). EPs involving nociception are also substantially modulated by participant variables including vigilance, emotional state, alertness and especially, attention to the stimulus (in review, Legrain et al., 2012). Conversely, the last decade of research produced data showing that the amplitudes of nociception-related EPs are largely independent of these psychophysiological conditions (Legrain et al., 2011, 2012; Torta et al., 2012; Ronga et al., 2013; Moayedi et al., 2015). Because of the classical principle in which the vertex potential reflects relevant sensory stimuli (Walter, 1964; Carmon et al., 1976), authors previously hypothesized that nociceptive EP waves of the vertex potential represent potential threats. Although this theory is still debated, because significant differences were observed between tSMS over M1 and that over S1/sham stimulation immediately after intervention, the decrease in IES-EP amplitude may not have been caused by changes in psychophysiological conditions, such as attenuation, habituation, or fatigue resulting from repetitive measurements. In addition, the stability of IES-EP amplitudes before tSMS in all stimulus conditions and immediately after and 10 min after tSMS over S1 and sham stimulation as seen in the current study may indicate that the participant’s attentiveness remained constant and that confounding factors were fairly well controlled. Although the focus of tSMS was small, we previously showed that various SEP components decrease according to the site of tSMS stimulation. For example, tSMS over C3 affects the N20 component of SEPs (Kirimoto et al., 2014), whereas the amplitude of N33 is modulated by tSMS over M1 (Kirimoto et al., 2016). Hence, we consider that the IES-EPs recorded in this study were robust and indicated attenuation by tSMS over M1.

### Putative Mechanisms of the Effect of tSMS over M1 on IES-EPs

In a review of NIBS modulation of LEPs in healthy subjects, the authors stated that the strongest effect was a lower susceptibility to nociceptive brain responses by HF rTMS of M1 in both patients and healthy individuals. However, cathodal tDCS over M1 reduces evoked pain more effectively than anodal tDCS, opposite of observations with spontaneous chronic pain (Mylius et al., 2012). In addition, M1 is generally considered the only validated target for modulation of nociceptive processing by cortical stimulation (Cruccu et al., 2016). HF rTMS over M1 reduces LEP amplitudes in healthy controls and patients with migraine, with the latter group showing a more pronounced effect (de Tommaso et al., 2010). They suggested that the decreased LEP amplitudes were due to the interaction between the motor cortex and nociceptive regions. LEP amplitudes mainly involve cortical areas that subtend vertex LEPs, an idea that is consistent with the functional relationship that is present between M1 and the anterior cingulate cortex (ACC). The ACC generates LEPs (Kakigi et al., 1995; Kanda et al., 2000; Frot et al., 2007; Valentini et al., 2012) and IES-EPs (Inui et al., 2002; Mouraux et al., 2010; Omori et al., 2013) along with the operculo-insular cortex and part of the salience network (Seeley et al., 2007; Menon and Uddin, 2010; Menon, 2015), which modulates multiple complex brain functions, including communication, social behavior, and self-awareness by integrating sensory, emotional, and cognitive information. Further, both Terney et al. (2008) and Csifcsak et al. (2009a) showed that cathodal tDCS over M1 reduces LEP amplitudes in healthy individuals. Both groups suggested that cathodal tDCS over M1 may provide secondary inhibition of the ACC, and hence, decrease LEP amplitude, because cathodal tDCS decreases regional cerebral blood flow in the right ACC and right thalamus, and because the ACC has wide projections with primary and premotor areas (Lang et al., 2005). These findings in which facilitatory HF rTMS and inhibitory cathodal tDCS share common analgesic effects are apparently contradictory. One possible explanation for this discrepancy is that the pain-relieving function of M1 does not involve motor corticospinal output processes, and because cathodal tDCS may also deactivate inhibitory M1 interneurons or inhibitory projections to the ACC (Lefaucheur et al., 2008, 2014, 2017; Mylius et al., 2012).

Another hypothesis explains why M1 is the most widely used target in experimental pain studies. Lefaucheur et al. (2006) proposed that restoration of SICI in the M1 induced by HF rTMS may have an analgesic effect. This could be indirectly and partly compatible with the result of our study in which tSMS over M1 exerted analgesic effects via brain responses to IES. In our previous studies, we demonstrated that 10 min of tSMS over M1 enhances SICI (Nojima et al., 2015, 2016), although we did not test this in the current study. In chronic neuropathic pain studies, HF rTMS and anodal tDCS of M1 restore SICI and are correlated with the amount of induced pain relief (Lefaucheur et al., 2006, 2012b; Antal et al., 2010; Mhalla et al., 2011). In addition, HF rTMS over M1 reduces the amplitudes of LEPs in parallel with laser-induced pain scores in patients with chronic neuropathic pain (Lefaucheur et al., 2010). Thus, the pain-relieving effects following M1 stimulation may be at least partly due to reestablishment of defective intracortical inhibitory processes (Lefaucheur et al., 2006, 2012b; Naro et al., 2016). A recent systematic review and meta-analysis of 43 studies with a combined total of 1009 patients with chronic pain and 658 healthy controls concluded that the extent of SICI is decreased, and short-interval intracortical facilitation is increased in patients with chronic pain compared with healthy individuals (Parker et al., 2016). These results indicate that chronic pain is associated with functional maladaptive plastic changes in M1, as well as in the so called “Salience network”, such as the S1, operculo-insular cortex, ACC, and thalamus. The reasons why facilitatory HF rTMS over M1 increases SICI in patients with chronic pain are unknown, and further studies are needed. In line with these studies, we speculate that inhibitory modulation of M1 by tSMS, especially the enhancement of SICI that we demonstrated in our previous studies (Nojima et al., 2015, 2016), is related to some aspects of nociceptive processing used in the generation of EPs in this study.

On the other hand, tSMS applied over S1 had no remarkable effect on IES-EPs in this study, whereas the amplitudes of LEPs are reportedly reduced following both (facilitatory) continuous TBS and (inhibitory) intermittent TBS over S1 (Poreisz et al., 2008), as well as cathodal tDCS over S1 (Antal et al., 2008). The authors of the previous reports speculated that when S1 is inhibited, the activity of the pain-related cortical network is reduced because of the extensive connections between S1 and other cortical regions, which could be the possible origin of LEPs, as was reported in studies on rTMS and tDCS over M1. Although we demonstrated the direct functional effects of tSMS over S1, remote effects on unstimulated areas were not confirmed in our study. Therefore, tSMS over S1 did not seem to modulate other areas that are estimated to be generators of nociceptive stimulation-related EPs in the current study.

### Dissociation between Perception and IES-EPs

Our behavioral data suggest that tSMS over M1 does not exert—at least with the stimulation parameters we used—a significant effect on sensory threshold, as was indicated by the fact that VAS scores of perceived pain sensations did not seem to reflect the pain reducing effects of tSMS over M1. Indeed, numerous studies have demonstrated close coupling between the amplitudes of LEPs and the intensity of pain perception (reviewed by Legrain et al., 2011, 2012). However, nociception is not identical to pain, which is a conscious experience. Nociception can produce responses in the brain in the absence of sensation of pain, as was seen during activation of the operculo-insular cortex by laser stimulation of anesthetized monkeys (Baumgärtner et al., 2006) and unperceived laser stimulation of humans with emerging pain (Lee et al., 2009). Moreover, in the “thermal grill illusion”, conditioning facilitates nociceptive EPs independently of reported unpleasantness (Jutzeler et al., 2017). In NIBS intervention studies, anodal and cathodal tDCS modulate cortical nociceptive processing in functional magnetic resonance imaging (Ihle et al., 2014) and magnetoencephalograms (Nakagawa et al., 2017) in a polarity-dependent manner, but have little to no impact on pain perception. In addition, the last decade of research has shown that the relationship between the magnitude of LEPs and the intensity of pain perception can be easily disrupted (Iannetti et al., 2008; Valentini et al., 2011; Torta et al., 2012). Three repeated nociceptive stimuli at a short, constant ISI substantially decrease the magnitude of nociceptive EPs without changing pain intensity. Thus, nociceptive EPs may not reflect cortical activity that is directly involved in pain perception, but rather may indicate processes that play a role in attention towards relevant stimuli (Legrain et al., 2011; Torta et al., 2013). In line with these interpretations regarding the dissociation between pain perception and the amplitude of nociceptive EPs, in this study, tSMS over M1 may have modulated cortical nociceptive responses, but not pain processing. Further clinical studies using higher IES stimulation intensities to activate pain processing pathways and studies with higher intensity or longer tSMS application are warranted.

In summary, important differences likely exist in the patterns and mechanisms of analgesia due to cortical stimulation between acute pain induced in healthy individuals and patients with chronic pain, and the results of evaluation of NIBS in subjects with experimental pain cannot be directly translated into the clinical treatment of pain (Lefaucheur et al., 2008, 2014, 2017; Mylius et al., 2012). Our result in which the brain response to Aδ fiber stimulation, as by IES-EPs of very low intensity, was modulated by tSMS over M1 possibly by enhancement of intracortical inhibitory circuits may open a new chapter in terms of NIBS modulation of nociceptive processing. Future studies must look more carefully into whether tSMS over M1 can enhance SICI and diminish short-interval intracortical facilitation in correlation with reduction of the amplitude of IES-EPs. The NdFeB magnet is an inexpensive industrial product that is easily available, and application of a magnet on the scalp does not require high operational skill compared to other conventional NIBS methods. Hence, tSMS may become an effective tool in home medical treatment or rehabilitation and may facilitate the treatment of various neurological disorders. Our observations in which tSMS over M1 affected cortical nociceptive processing suggest that tSMS may function as a new NIBS tool for treatment of chronic pain in combination with conventional NIBS methods.

## Author Contributions

HK, HT, HO, IN and AO conceived of the study and designed the experimental paradigm. HK, NO and KY performed the experiments and analyzed the data. HK wrote the manuscript. HT, HO, IN and AO provided feedback and edited the manuscript. All authors read and approved the final manuscript.

## Conflict of Interest Statement

The authors declare that the research was conducted in the absence of any commercial or financial relationships that could be construed as a potential conflict of interest.
